# Immune analysis according to Lauren type for gastric cancer and its significance in individual treatment and prognostic prediction

**DOI:** 10.3389/fimmu.2025.1589513

**Published:** 2025-07-24

**Authors:** Huiwen Lu, Junqiao Yao, Chi Xue, Rui Huang, Baojun Huang

**Affiliations:** ^1^ Department of Anal and Intestinal Surgery, Tianjin Union Medical Center, The First Affiliated Hospital of Nankai University, Tianjin, China; ^2^ Department of Surgical Oncology and General Surgery, The First Hospital of China Medical University, Shenyang, China; ^3^ Key Laboratory of Precision Diagnosis and Treatment of Gastrointestinal Tumors (China Medical University), Ministry of Education, Shenyang, China

**Keywords:** gastric cancer, Lauren types, immunosenescence, immune analysis, prognosis, risk model

## Abstract

**Background:**

Numerous studies have proved that Lauren types are associated with the prognosis of gastric cancer patients. Whereas their associations with tumor immunity and significance in treatment remain unclear.

**Method:**

Eligible patients with gastric cancer (GC) who underwent curative resection at our institution from 2017 to 2023 were identified for this study. Tumor specimens were collected and processed with immunohistochemical staining to detect the difference in immune marker expression between Lauren types. Additional GC data related to human subjects were collected from GEO public dataset. Further analysis was then performed using TCGA and GEO datasets. GC patients in public datasets were divided into two groups according to their Lauren types. Survival analysis was performed between subtypes. The differences in infiltrating immune cells, human leukocyte antigen (HLA) family, checkpoints, and apoptosis-regulated genes between groups were analyzed. Then, associations between Lauren types and clinicopathological features were analyzed.

**Results:**

GC patients with diffuse type showed higher expression of CD3 (P=0.042), CD8 (P=0.025), and CD57 (P=0.020) then those with intestinal types. Among 300 GC patients in the GEO training set, patients with diffuse type showed a poorer prognosis than intestinal-type ones (OS: P<0.001; RFS: P=0.005). The diffuse subtype had more immune cells but was less functional than the intestinal subtype. Notably, checkpoints were highly expressed among diffuse-type patients. Intestinal patients had a higher positive rate of HER2 than diffuse ones. To find the hub genes, a three-gene-included risk model based on Lauren was constructed. The risk score was independently associated with survival of gastric cancer patients, regardless of OS and RFS (HR for OS: 2.517, 95% CI: 1.236-5.126; HR for RFS: 3.469, 95% CI: 1.644-7.321). ROC analysis showed that this risk model had a good predictive ability. High-risk patients had more advanced T (P<0.001), M (P<0.05), and pathological stage (P<0.001), indicating that those with the high risk presented more aggressive features. Immune analysis was consistent with Lauren type. Results from the TCGA validation group were consistent with the GEO training set.

**Conclusion:**

Diffuse-type tumors exhibited greater immune cell abundance but reduced functional activity, contributing to poorer prognosis. These tumors also demonstrated potentially higher sensitivity to immunotherapy and chemotherapy compared to intestinal-type tumors. HER2-targeted therapy combined with chemoradiotherapy is strongly recommended for intestinal-type GC patients. These disparities are primarily attributable to upregulated LINC00702, C8orf88, and FILP1 in diffuse-type GC patients

## Introduction

Although gastric cancer (GC) had a declined incidence over the last decade due to the eradication of *Helicobacter pylori* (*H. pylori*) infections, it is still the third leading cause of cancer-related death (1). GC can be macroscopically classified based on its gross appearance, as defined by Borrmann. This classification method is still of great significance and has relevance with microscopic sorts. Microscopically, Lauren performed GC staging according to whether there was a glandular or tubular growth pattern (intestinal type) or not (diffuse type). Based on that, the biological differences of the tumor can be favorably reflected due to the fact that they cannot transfer from one into the other throughout tumor growth and metastasis (2). There are numerous differences between intestinal-type and diffuse-type GC. In etiology and pathogenesis, intestinal-type GC is correlated with dietary habits and environmental risk factors, while diffuse-type GC is closely related to genetic ones (1). Most intestinal-type patients are older males, while younger female patients are more prone to diffuse-type GC (3). Diffuse-type tumors prefer to spread in the upper layer of the stomach wall and invade the submucosa at an early stage, rather than penetrate the lumen, thus causing fibrosis and rapid tumor development (4). Some studies have revealed that diffuse-type GC patients may have a worse prognosis compared with intestinal-type ones due to the presence of more aggressive factors (3, 5, 6). It has been demonstrated that Lauren types are closely correlated with sensitivity to chemotherapy. Diffuse-type patients benefit more from chemotherapy, while intestinal-type patients may be more suitable to receive chemoradiotherapy (7–11). Thus, it is valuable to explore the role of Lauren subtypes in the progression of GC.

The tumor immune microenvironment (TIM) has attracted more attention in recent years with the development of immunotherapy. To our knowledge, the differential infiltration of tumor cell nests and intratumoral immune cells, such as T-lymphocytes, natural killer (NK) cells, and macrophages, reflects the immune status, and it can be employed to predict the therapeutic responses to chemotherapy or immunotherapy for GC patients. According to some studies, CD8+ T lymphocytes and NK cells are the main cytolytic effectors involved in immunosurveillance, and myeloid-derived suppressor cells (MDSCs) and regulatory T cells play critical roles in tumor development (12–15). Additionally, some immunosuppressive factors in the TIM, such as interleukin-10 (IL-10) and transforming growth factors-β1 (TGFβ-1), can be upregulated in tumors. Notably, the expression of immune checkpoints, like PD-L1, varies among different Lauren types (16, 17). The infiltration of immune cells in the TIM mentioned above differs with disease stages and hence it can provide guidance for treatment options.

However, there is still a lack of interpretation of the difference in the TIM between intestinal-type and diffuse-type GC. Besides, it remains unclear about the effects of Lauren types on guiding personalized treatment for GC patients. Pernot et al. revealed that the diffuse-type AGC as a “cold tumor” had a lower level of CD8+ TIL, NK, and Tregs than the intestinal-type one (18), Whereas, Li et al. demonstrated that there were more abundant but less functional intramural CD8+ TILs (19). The focus of most of these studies is placed on specific immune cells, and consistent conclusions cannot be obtained due to potential selection bias and varied testing methods. In this study, tumor-infiltrating cells and immune functions were compared between two Lauren subtypes. In addition, a Lauren-related-regulated model was established to predict the prognosis of GC patients and provide potential therapeutic targets. Moreover, an analysis was also performed based on Gene Expression Omnibus (GEO) and The Cancer Genome Atlas (TCGA) databases. This in-depth analysis of immune differences between Lauren subtypes enhances understanding of molecular features in gastric cancer (GC), potentially advancing precision treatment strategies for GC patients

## Materials and methods

### IHC staining

A total of 360 GC patients who undergone curative surgery in the First Affiliated Hospital of China Medical University between 2017 to 2023 were included in this study. Inclusion criteria were (1): histologically confirmed primary advanced gastric adenocarcinoma (2); curative resection with D2 or more extensive lymphadenectomy (3); tumor specimens collected and fixed in 10% formalin within 30 minutes post-resection, followed by paraffin embedding (4); availability of complete postoperative clinicopathological reports. Exclusion criteria comprised (1): clinical or radiological evidence of distant metastasis or peritoneal dissemination (2); history of other malignancies (3); receipt of neoadjuvant therapy (4); loss to follow-up or death within one month post-surgery. The study was proved by the Institutional Review Board of the Ethics Committee of China Medical University.

The deparaffinized GC specimens were dehydrated with ethanol, and then incubated with hydrogen peroxide for 30 min to make the endogenous peroxidase inactivated. The non-specific binding sites were blocked with bovine serum albumin. Following that, sections were exposed to primary antibodies overnight against CD45, CD8, CD3, CD20, CD57, CD68, CD66, PD-1 and HER2 respectively. An additional 15 min for cleaning the primary antibodies was carried out before exposure to the secondary antibodies for 30 min. Immunoreactivity can be evaluated after exposure to the DAB chromogen for 5–10 min. Finally, the processed specimens were counterstained with hematoxylin, dehydrated and mounted.

### Immunoreactive scores

Two investigators (Lu HW and Huang R) evaluated the IHC staining levels independently and compared their results. Another investigator (Huang BJ) re-examined the controversial results. Finally, a consensus score was achieved for each tumor specimens. Investigators evaluated the different staining densities and calculated the proportion of positively-stained ones among all cells for each processed specimen in four high-power magnification fields (x200) with an average of 1000 cells. Staining intensity (SI) was evaluated as following: no staining = 0; light yellow (weak staining) = 1; bright yellow (moderate staining) =2; and brown (strong staining) =3. Percentage of positively-stained cells (PP) was divided into four groups: positively-stained cells<5%=0; 5%-25% positively-stained cells =1; 26%-50% positively-stained cells=2; 51%-75% positively-stained cells = 3; and positively-stained cells>75% = 4. Immunoreactive score (IRS) was determined by multiplying SI by PP. The degrees of CD45, CD8, CD3, CD20, CD57, CD68, CD66, PD-1 and HER2 for each specimen were classified respectively as low expression group (IRS < 4) and high expression group (IRS >=4).

### Data collection and procession

Gastric cancer RNA-seq data and clinical information were downloaded from the GEO database (GSE62254: https://www.ncbi.nlm.nih.gov/geo/). Validating data set was obtained from the TCGA database (https://portal.gdc.cancer.gov/). Only gastric cancer samples with complete clinicopathological data and survival data in data sets can be included in this study.

### Lauren type-based classification

GC patients in the training dataset were grouped based on Lauren type. Kaplan- Meier curve and log-rank test were performed to analyze the survival difference between groups. P-value <0.05 was considered significant. CIBERSORT algorithm was used to evaluate the proportions of infiltrated immune cells. Additionally, we used the ESTIMATE package to measure the stromal, immune, and estimate scores for every patient, which quantified the stromal cells, immune cells, and tumor purity, respectively. The treatment efficiency of immunotherapy for groups was evaluated by comparing checkpoints’ expression. Furthermore, the expression of apoptosis-related genes was evaluated in subgroups to estimate cell death.

### Screening for differentially expressed genes

The Lauren-based model was constructed to prove its predictive significance for GC patients. Batch normalization between the training set and validating set was performed before the construction and validation of the risk model to be comparable. Genes obtained from GEO and TCGA data sets were intersected to identify the genes presented in both. Then, genes differentially expressed in Lauren-based groups were screened within the GEO data set using the limma package in R software (version 4.0.4). Adjusted P value <0.05 was considered significant. Results were shown as a heat map created by the pheatmap package in R software. In addition, we performed Gene Ontology (GO) and Kyoto Encyclopedia of Genes and Genomes (KEGG) to explore the enrichment of these DEGs in biological function and signaling pathways (20).

### Construction and validation of Lauren-related regulators signature

Lauren-related DEGs were identified between groups ((|logFC|>1.5, adjusted *P<*0.05), and then univariable COX regression was performed to screen the genes which were associated with overall survival (OS) (P<0.05). Later, lasso regression analyzed prognosis-related DEGs to decrease the false positives in variables. Notably, only genes with non-zero coefficients were used to construct an optimal predictive model and calculate the risk scores for patients. The calculation of risk score for each patient were detailed description in **Supplementary Materials**.

### Association between Lauren-based gene signature and clinicopathological characteristics

Patients’ clinicopathological characteristics included sex, age, peritoneal and lymphatic invasion, T, N, and M stage, pathological TNM stage, numbers of positive lymph nodes, and tumor location, downloaded from GEO TCGA datasets. T-test or one-way ANOVA was used to compare the continuous variables between groups, and categorical variables were analyzed by Fisher’s exact test or chi-square test. Univariant and multivariant Cox regressions were performed to determine the independent prognostic factors for gastric cancer patients, and the hazard ratio (HR) and its 95% confidence interval (CI) were estimated. Considering that risk score was measured according to Lauren classification, Lauren type was not included in COX regression to avoid multi-collinearity bias. SPSS 22.0 statistical software was used to perform the analyses above. Immune analysis was performed using CIBERSORT algorithm and ESTIMATE packages.

### Gene set enrichment analysis and development of a predictive nomogram

GSEA was performed to compare the different activated signaling pathways between low- and high-risk groups, and a relative plot was shown. Meanwhile, the nomogram model was built based on prognosis-related clinicopathological characteristics and risk scores to predict the survival of GC patients more accurately. The nomogram model included variables screened by univariant and multivariant COX regression analysis. 1-year, 3-year, and 5-year calibration curves were plotted to evaluate predicted and actual survival differences. Additionally, 1-year, 3-year, and 5-year ROC curves were performed by survival ROC package to estimate the accuracy of survival prediction of the nomogram model.

### Statistical analysis

R software (version 4.0.4) and SPSS 22.0 were used for data analysis. Overall survival (OS) and recurrence-free survival (RFS) with 95% CI were survival endpoints. The prediction was acceptable when the area under the curve (AUC) value was higher than 0.6 (95% CI: 0.5-0.7). A P-value less than 0.05 was considered to be statistically significant. The figures of COX regression were completed using GraphPad Prism 8.

## Results

### IHC staining of CD3, CD8, and CD57 were associated with Lauren types of GC

**Figure 1:**
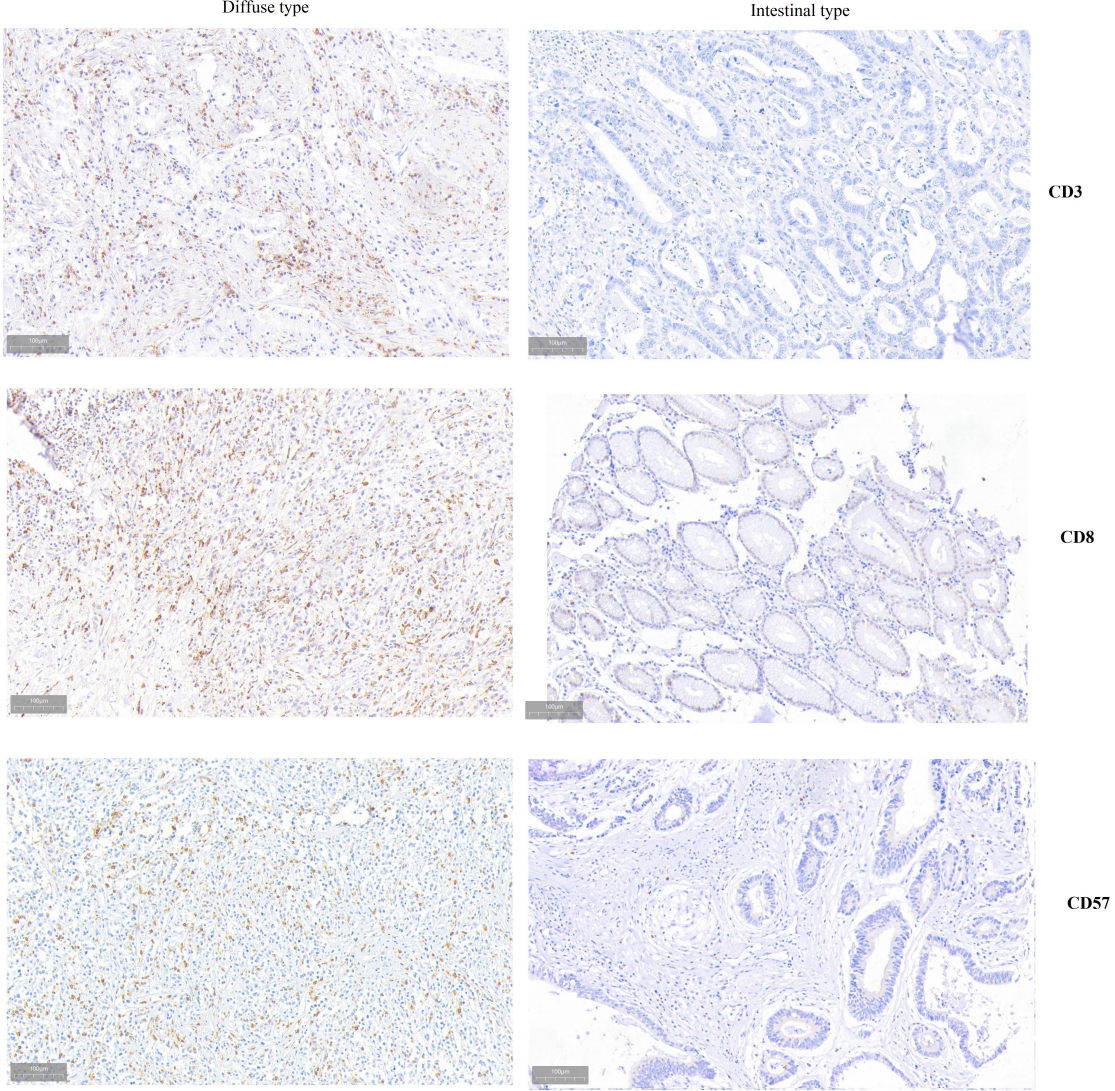
Differences of IHC staining patterns between Lauren types.

Through comparing the IHC staining patterns of CD45, CD8, CD3, CD20, CD57, CD68, CD66, PD-1 and HER2 between Lauren types, it can be found that diffuse type had higher expression of CD3 (P-0.042), CD8 (P=0.025), and CD57 (P=0.020) than intestinal types (**Figure 1**, **Table 1**). The IHC staining patterns of CD45, CD20, CD68, CD66, PD-1 and HER2 were similar between Lauren types.

**Table 1:** Associations between Lauren type and IHC staining patterns.

Variable	Lauren Types			P value
	All patients	Intestinal	Diffuse	
CD57	344	161	183	
High expression	267 (77.6%)	116 (72.0%)	151 (82.5%)	0.020
Low expression	77 (22.4%)	45 (28.0%)	32 (17.5%)	
CD3	352	161	191	
High expression	267 (75.9%)	114 (70.8%)	153 (80.1%)	0.042
Low expression	85 (24.1%)	47 (29.2%)	38 (19.9%)	
CD45	313	144	169	
High expression	163 (52.1%)	70 (48.6%)	93 (55.0%)	0.257
Low expression	150 (47.9%)	74 (51.4%)	76 (45.0%)	
CD8	350	161	189	
High expression	255 (72.9%)	108 (67.1%)	147 (77.8%)	0.025
Low expression	95 (27.1%)	53 (32.9%)	42 (22.2%)	
CD20	327	149	178	
High expression	246 (75.2%)	109 (73.2%)	137 (77.0%)	0.426
Low expression	81 (24.8%)	40 (26.8%)	41 (23.0%)	
CD66	330	149	181	
High expression	191 (57.9%)	81 (54.4%)	110 (60.8%)	0.240
Low expression	139 (42.1%)	68 (45.6%)	71 (39.2%)	
CD68	278	132	146	
High expression	80 (28.8%)	42 (31.8%)	38 (26.0%)	0.287
Low expression	198 (71.2%)	90 (68.2%)	108 (74.0%)
PD-1	325	150	175	
High expression	172 (52.9%)	71 (47.3%)	101 (57.7%)	0.062
Low expression	153 (47.1%)	79 (52.7%)	74 (42.3%)	
ERBB2	336	154	182	
High expression	54 (16.1%)	21 (13.6%)	33 (18.1%)	0.264
Low expression	282 (83.9%)	133 (86.4%)	149 (81.9%)	

### Factors associated with Lauren types and survival analysis

**Figure 2:**
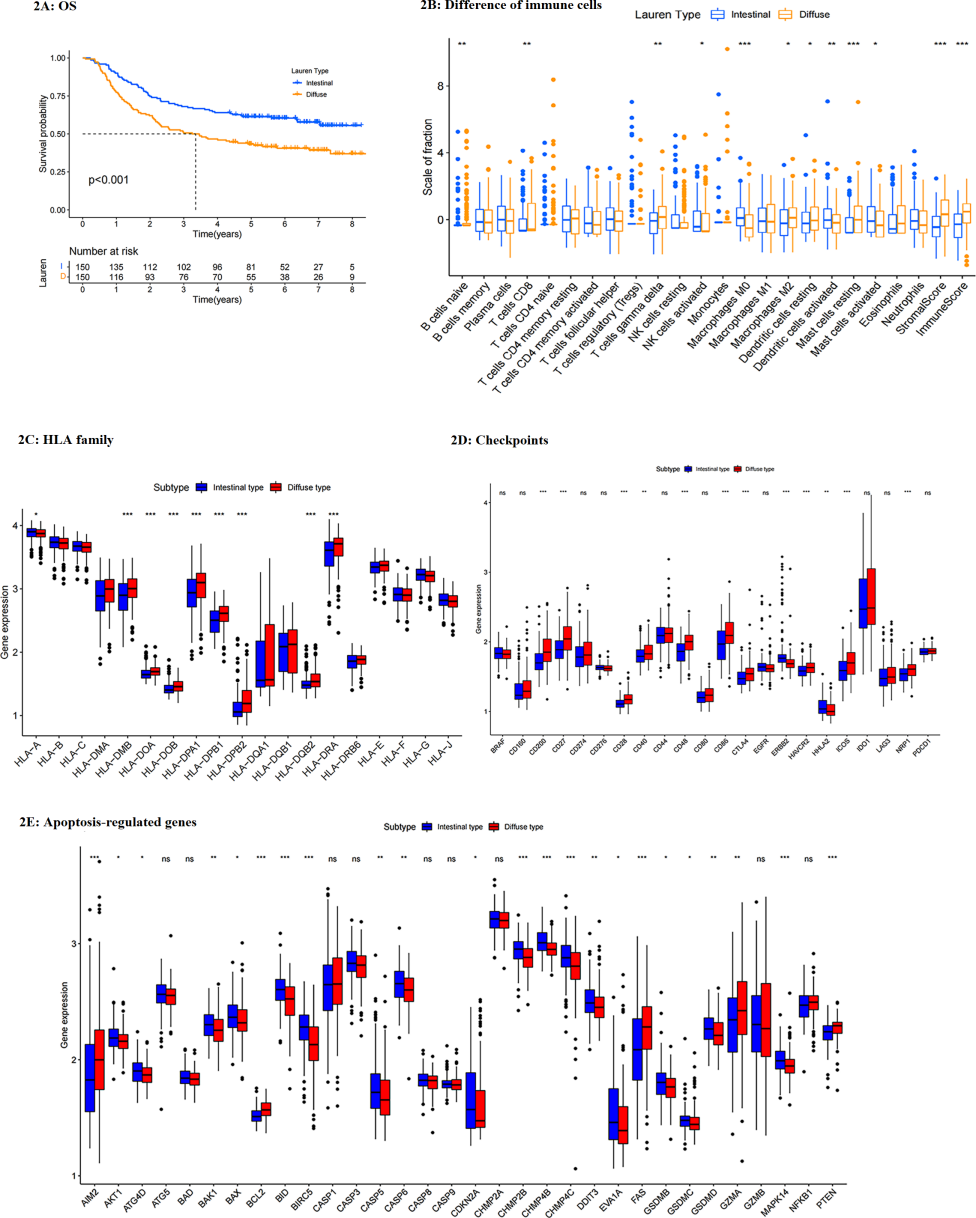
**(A)** Survival differences between intestinal- and diffuse- type patients (OS, training set); **(B)** difference in the expressions of immune cells between Lauren subtypes. **(C)** difference in HLAs between Lauren subtypes; **(D)** difference in checkpoints between Lauren subtypes; **(E)** difference in apoptosis-regulated genes between Lauren subtypes. ***: p<0.001,**: p<0.01,*: p<0.05.

According to Lauren types, three hundred gastric cancer patients in the GEO training dataset were classified into two groups. Since mixed type presented similar survival with diffuse type among advanced GC patients, and only eight patients were mixed type in the training set, mixed-type patients were classified into the diffuse group. One hundred fifty patients were in the intestinal group and 150 in the diffuse group. Through K-M analysis, it could be found that intestinal-type patients had better survival than diffuse-type ones, regardless of OS or RFS (RFS, 1-y: 80.7% vs 67.3%, 3-y: 63.7% vs 47.6%, 5-y: 59.8% vs 43.5%, P=0.005; OS, 1-y: 89.3% vs 77.3%, 3-y: 68.0% vs 51.3%, 5-y: 61.3% vs 42.4%, P<0.001) (**Figure 2A**, **Supplementary Figure S1a**). The associations between Lauren types and clinicopathological characteristics were shown in **Table 2**. It could be found that young female patients were more likely to suffer diffuse-type gastric cancer (P<0.001). The diffuse-type tumor invaded the peritoneum and lymphatic vessel more easily than the intestinal one (P=0.002 and P=0.004, respectively). Additionally, diffuse-type patients had more metastatic lymph nodes (P=0.001). Notably, GC patients with diffuse-type tumors showed more advanced T (P<0.001), N (P<0.001), M stage (P=0.004), and pathological stage (P<0.001). Thus, diffuse-type patients presented with more advanced pathological stages and aggressive features, explaining their poorer prognosis relative to intestinal-type patients. Younger females were more likely to suffer diffuse-type GC.

**Table 2:** Associations between Lauren type and clinicopathological features.

Variable	Lauren Types			P value
	All patients (N=300)	Intestinal (N=150)	Diffuse (N=150)	
Age, Average, years	61.94 (24–86)	64.45 (31–86)	59.43 (24–84)	<0.001
Gender, N (%)				
Female	101 (33.7%)	36 (24.0%)	65 (43.3%)	<0.001
Male	199 (66.3%)	114 (76.0%)	85 (56.7%)	
Peritoneal invasion				
Yes	88 (35.6%)	36 (27.1%)	52 (45.6%)	0.002
No	159 (64.4%)	97 (72.9%)	62 (54.4%)	
Venous invasion				
Yes	44 (25.4%)	26 (27.1%)	18 (23.4%)	0.603
No	129 (74.6%)	70 (72.9%)	59 (76.6%)	
Lymphatic vessel invasion				
Yes	205 (73.7%)	93 (66.0%)	112 (81.8%)	0.004
No	73 (26.3%)	48 (34.0%)	25 (18.2%)	
Number of examined LN, Average	38.97 (10–142)	37.53 (10-142)	40.42 (12-94)	0.632
Number of positive LN, Average	8.45 (0-61)	6.33 (0-40)	10.58 (0-61)	0.001
Location				
Antrum/pylorus	163 (54.3%)	82 (54.7%)	81 (54.0%)	0.487
Body/fundus	107 (35.7%)	56 (37.3%)	51 (34.0%)	
GEJ/cardia	30 (10.0%)	12 (8.0%)	18 (12.0%)	
Pathological T stage				
T2	186 (62.4%)	111 (75.0%)	75 (50.0%)	<0.001
T3	91 (30.5%)	26 (17.6%)	65 (43.3%)	
T4	21 (7.0%)	11 (7.4%)	10 (6.7%)	
Pathological N stage				
N0	38 (12.7%)	28 (18.7%)	10 (6.7%)	<0.001
N1	131 (43.7%)	73 (48.7%)	58 (38.7%)	
N2	80 (26.7%)	32 (21.3%)	48 (32.0%)	
N3	51 (17.0%)	17 (11.3%)	34 (22.7%)	
Pathological M stage				
M0	273 (91.0%)	144 (96.0%)	129 (86.0%)	0.004
M1	27 (9.0%)	6 (4.0%)	21 (14.0%)	
Pathological TNM stage				
I	30 (10.0%)	23 (15.3%)	7 (4.7%)	<0.001
II	97 (32.3%)	59 (39.3%)	38 (25.3%)	
III	96 (32.0%)	40 (26.7%)	56 (37.3%)	
IV	77 (25.7%)	28 (18.7%)	49 (32.7%)	

### Differences in immune functions between groups

Through ESTIMATE analysis, diffuse-type tumors performed higher stromal scores, immune scores, and total scores, representing that diffuse groups had more stromal cells and immune cells but lower tumor purity than intestinal ones (**Supplementary Figure S1b**). Although diffuse-type tumors had a higher immune score, the immune analysis showed that immune cells activated by diffuse tumors were mostly immune-inhabited effectors, resting cells, or tumor-promoted effectors, such as naïve B cells, M0 and M2 macrophages, resting dendritic cells (DC), and resting mast cells. In contrast, those cells which improved immunity were less activated in the diffuse group, including activated nature kill (NK) cells, activated DCs, and activated mast cells (**Figure 2B**). In comparison, diffuse-type patients showed more abundant CD8 T cells, which was potentially the cause of their lower tumor purity.

In addition, through human leukocyte antigen (HLA) analysis, it could be found that most HLA-II molecules, which were responsible for antigen recognition of immune cells, were highly expressed in diffuse-type tumors (**Figure 2C**). It can be speculated that highly expressed HLA-II was the possible cause of more abundant CD8 T cells and higher TME scores in the diffuse group than in the intestinal one. Next, we compared the expression of typical checkpoints between groups. It indicated that most checkpoints were increased in diffuse groups, including CD200, CD27, CD28, CD40, CD48, CD86, CTLA4, HAVCR2, IDO1, and NRP1, while ERBB2 and HHLA2 were highly expressed in the intestinal group (**Figure 2D**). Thus, most immunotherapy could be suggested for diffuse-type patients, while HER-2 targeted therapy was more suitable for intestinal patients.

Then, we compared the expression of apoptosis-regulated genes between groups (**Figure 2E**). Results showed that most apoptosis- or autophagy- regulated genes were reduced expressed in diffuse-type tumors, indicating that diffuse-type gastric cancer had more activated tumor cells than intestinal-type GC. To be concluded, diffuse-type GC had more abundant immune cells but fewer functions than the intestinal tumor. Furthermore, the diffuse-type tumor showed an anti-apoptotic feature, resulting in its more aggressive characteristic.

### Screening for Lauren-related DEGs

Through limma analysis, 13 genes were significantly upregulated in diffuse-type GC patients (**Supplementary Table S1**). The expression of these Lauren-related DEGs was shown as a heatmap in **Supplementary Figure S2a**. Through GO analysis, we found that these DEGs played a vital role in regulating intracellular physiological processes and biological functions (**Supplementary Figure S2c**). KEGG analysis showed that these Lauren-related DEGs regulated cell adhesion and enriched tumor-promoted pathways, including chemokine signaling and cGMP-PKG pathways (**Supplementary Figure S2e**).

### Establishment of prognostic gene signature based on Lauren types

**Figure 3:**
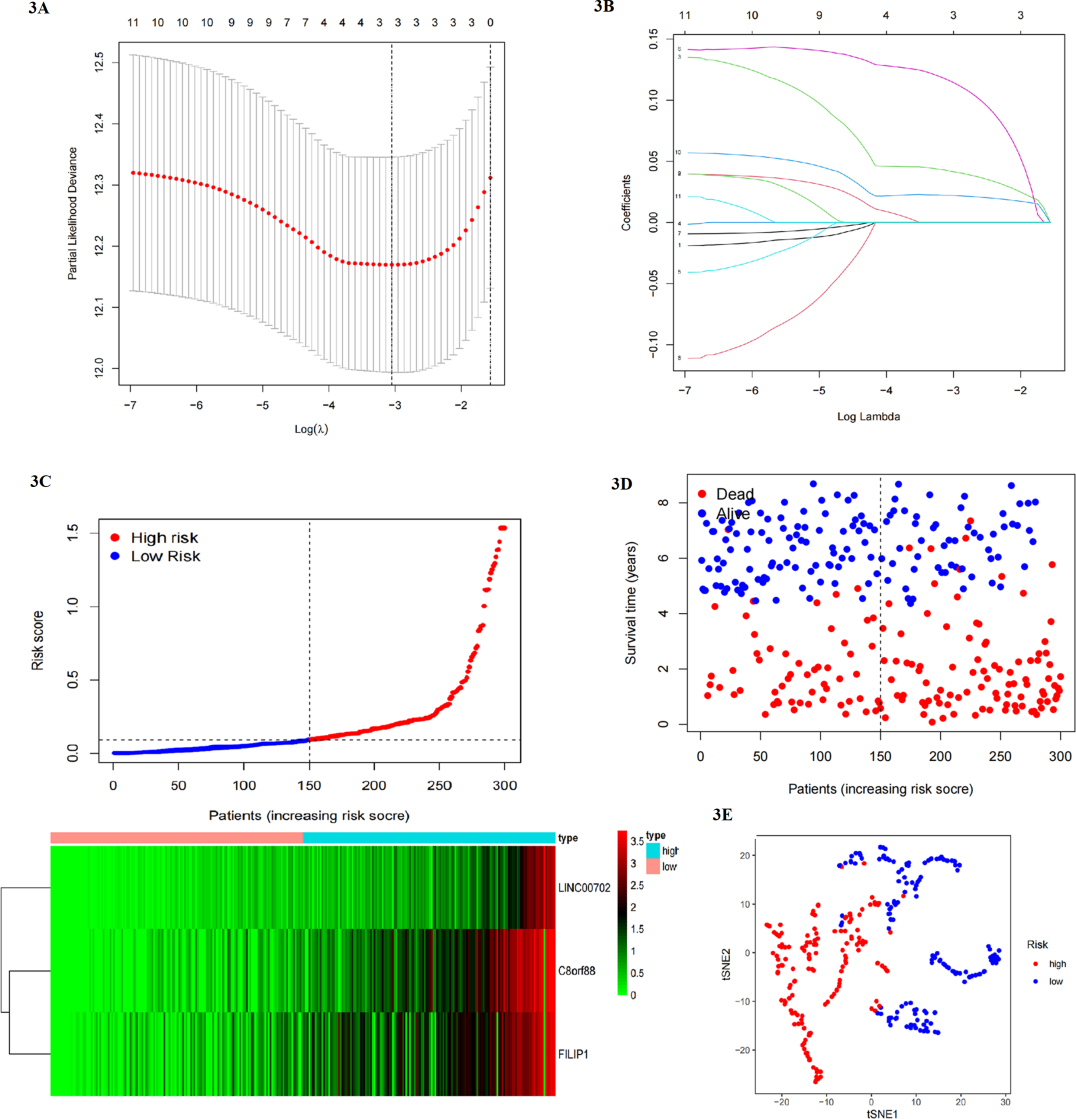
**(A, B)** Lasso regression to eliminate genes closely correlated with others. **(C)** GC patients with low risk had a longer survival time than those with high risk, and genes were upregulated in high-risk group. **(D, E)** patients could be divided into two clusters by risk model (blue ones and red ones).

Univariable Cox regression was used to identify the genes associated with prognosis. Eleven genes were screened and shown in **Supplementary Figure S2b**. Then, LASSO regression was analyzed to eliminate genes closely correlated with others. After 1000 resamples, a 3-gene prognostic risk model was constructed (**Figures 3A, B**), including C8orf88, LINC00702, and Filamin A Interacting Protein 1 (FILIP1). GC patients with low risk had a longer survival time than those with high risk, and the heap map was shown in **Figure 3C**. Risk scores were calculated for each patient: risk score = C8orf88 expression * 0.041919 + LINC00702 expression * 0.115662 + FILIPI expression * 0.022185. The median score of the GEO training cohort was regarded as the cut-off value. Patients in GEO and TCGA cohorts were divided into low- and high-risk groups using the same cut-off value (GEO cohort: **Figure 3D**). The PCA revealed that GC patients were well separated into two clusters (**Figure 3E**).

### The difference in prognosis and signaling pathways between two risk groups and functional analysis

**Figure 4:**
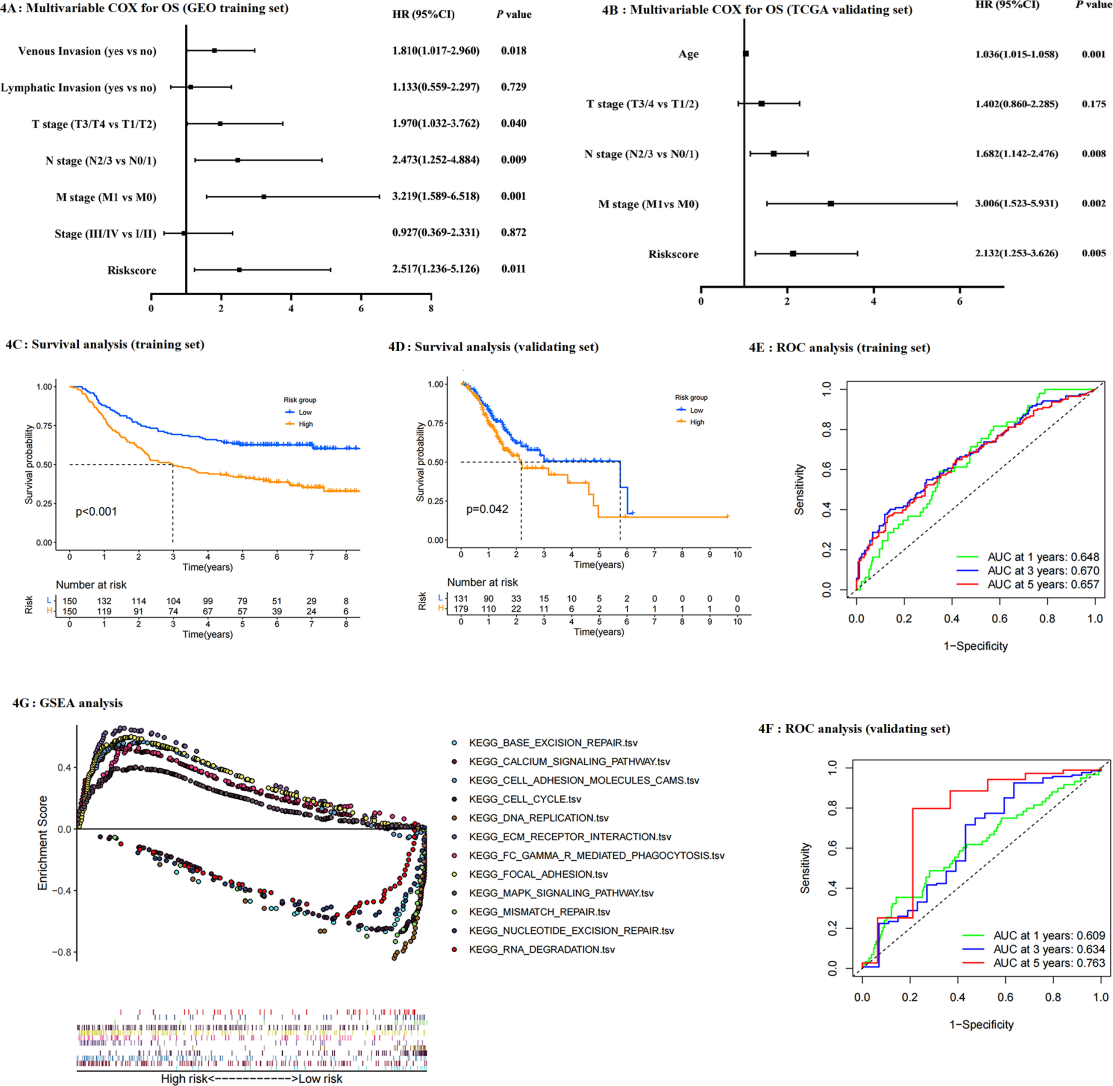
**(A, B)** Multivariable COX regression for OS using training set and validating set respectively; **(C, D)** survival difference (OS) between risk groups (training set: **C**; validating set: **D**); **(E, F)** ROC to analyze the predictive efficiency of risk model (training set: **E** validating set: **F**); **(G)** GSEA pathway analysis based on risk model.

Survival analysis showed that patients in the high-risk group had a poorer prognosis than those in the low-risk group (OS and RFS: P<0.001 in the training set) (**Figure 4C**, **S3a**). In addition, we performed univariable and multivariable COX regression analysis, which included clinicopathological factors and risk score, to estimate whether the risk model could be independently predictive for survival of gastric cancer patients. Results indicated that venous invasion (HR for OS:1.810, 95% CI: 1.017-2.960; HR for RFS: 2.049, 95% CI: 1.196-3.509), T stage (HR for OS: 1.970, 95% CI: 1.032-3.762; result of RFS was non-significant), N stage (HR for OS: 2.473, 95% CI: 1.252-4.884; HR for RFS: 2.491, 95% CI: 1.063-5.841), M stage (HR for OS: 3.219, 95% CI: 1.589-6.518; HR for RFS: 2.615, 95% CI: 1.162-5.889), and risk score (HR for OS: 2.517, 95% CI: 1.236-5.126; HR for RFS: 3.469, 95% CI: 1.644-7.321) were significantly associated with survival (**Figure 4A**, **S3b–d**). In addition, we perform GSEA to analyze the difference in signaling pathways between groups (**Figure 4G**). Results showed that the high-risk group activated tumor-related pathways, such as MAPK and calcium signaling pathways, and improved focal adhesion, which contributed to tumor development and metastasis, while the low-risk group presented activity in regulation and monitoring for intracellular physiological processes, such as cell cycle, metabolism, and mismatch repair. Next, the ROC curve evaluated the predictive efficiency of the Lauren-based prognostic model. 1-year, 3-year, and 5-year AUC values were 0.648, 0.670, and 0.657 respectively (**Figure 4E**). Thus, the risk model presented better predictive accuracy for the survival of gastric cancer patients.

### Validating the Lauren-related regulators signature

As mentioned above, gastric cancer patients in the TCGA validating dataset were classified as low- and high-risk groups. Univariable Cox (not shown in the study) and multivariable COX analysis, which included clinical traits and risk score, found that Lauren-related risk score was independently associated with OS (HR: 2.132, 95% CI: 1.253-3.636 **Figure 4B**). Notably, although the variables used in the multivariate COX analysis in the training dataset differed from those from the validating dataset, caused by the limited clinical information provided in the TCGA cohort, the risk score was still independently associated with survival. Thus, it can be concluded that the risk model was significant and practical. PCA and survival analysis were performed, respectively as shown in **Figure 4D** and **Supplementary Figure S4**. Results indicated that patients in the low-risk group had a better prognosis than those in the high-risk group (P=0.042). The 1-year, 3-year, and 5-year AUC values were 0.609, 0.634, and 0.763, respectively (**Figure 4F**). Surprisingly, the risk model presented superior predictive efficiency for long-term survival. Validating results were consistent with the GEO training set, thus it could be concluded that this predictive model was accessible for predicting the survival of gastric cancer patients, and Lauren type could potentially be practical in clinical diagnosis and guidance for treatment.

### Clinicopathological analysis according to risk model

**Figure 5:**
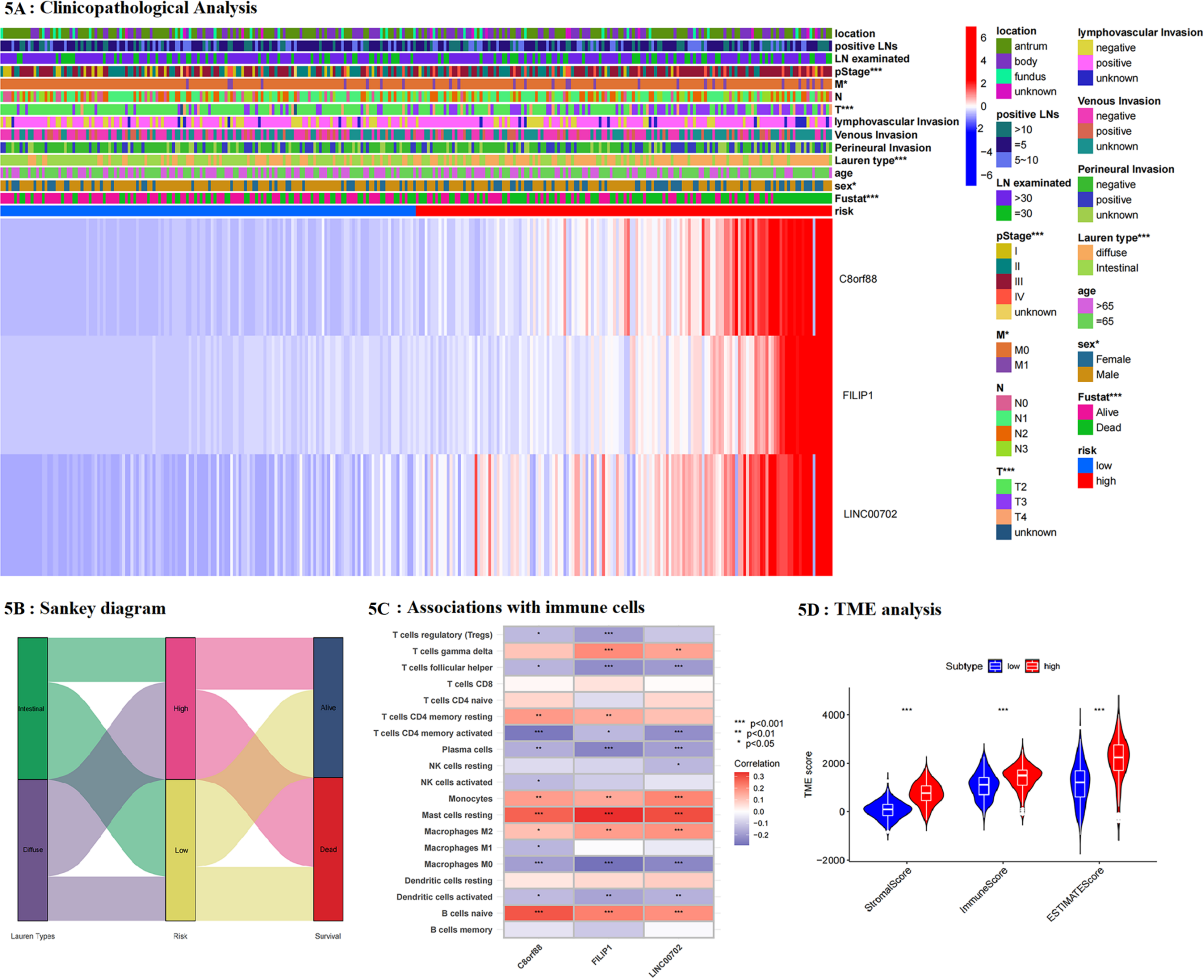
**(A)** The associations between clinicopathological features and risk groups; **(B)** Sankey diagram was performed to present the relationship among Laure types, risk groups and survival of patients; **(C)** the association between genes included in the risk model and immune cells; **(D)** tumor microenvironment (TME) analysis based on risk model.

The associations between risk groups and clinicopathological features were analyzed (**Figure 5A**). It noted that C8orf88, LINC00702, and FILIP1 were upregulated in the high-risk group, and this risk model was consistent with Lauren types. Clinicopathologic analysis revealed that female patients accounted for a larger proportion of the high-risk group, and patients with high risk had more advanced T, M, and pathological TNM stages. Thus, it could be concluded that GC patients with high risk had a more advanced pathological stage and poorer prognosis than those with low risk, potentially resulting from their aggressive features and increased activity in tumor-related pathways. Associations among Lauren type, risk model, and patients’ survival were presented as Sankey in **Figure 5B**.

### Comparison of the difference in immune activities between risk groups

**Figure 6:**
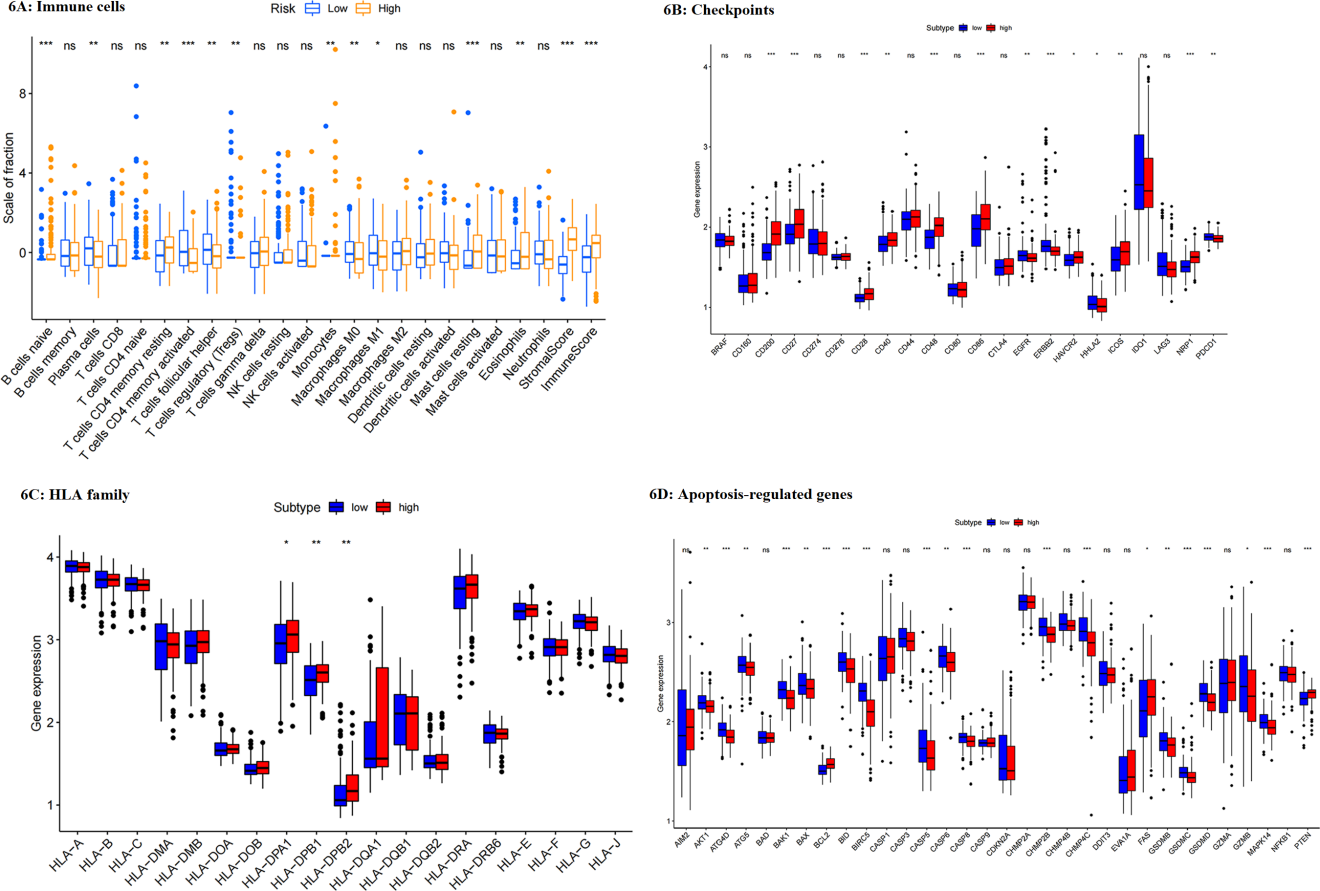
**(A–D)** Difference in the expressions of immune cells **(A)**, checkpoints **(B)**, HLAs **(C)**, and apoptosis-regulated genes **(D)**. ***: p<0.001,**: p<0.01,*: p<0.05.

In addition, we analyzed the association between genes included in the risk model and immune cells (**Figure 5C**). It noted that these genes were positively associated with tumor-promoted effectors, and those responsible for poor immunity, including M2 macrophages, resting CD4 T cells, resting mast cells, and naïve B cells, whereas they were negatively associated with the expression of helper T cells, activated CD4 T cells, plasma cells, M0/M1 macrophages and activated DCs, which were contributed to activated tumor immunity. Through ESTIMATE analysis, the high-risk group had higher stromal and immune scores than the low-risk group and a lower tumor purity, consistent with Lauren-based analysis (**Figure 5D**). The comparison of infiltrated immune cells between groups showed that resting and navies immune cells, and tumor-promoted effectors were upregulated in the high-risk group. In contrast, immune-activated or tumor-inhibited effectors, such as activated CD4 T cells, NK cells, plasma cells, M0, and M1 cells, were down-regulated (**Figure 6A**). Thus, it could be concluded that the high-risk group had more immune cells but fewer functions. Next, HLA expression was analyzed. It showed that high-risk patients had higher expression of HLA-II, including HLA-DR, HLA-DP and HLA-DQ, demonstrating that the high-risk group was better at antigen recognition (**Figure 6C**). Furthermore, we compared the expression of immune checkpoints between groups. Consistent with Lauren type, most checkpoints were highly expressed in the high-risk group, except for EGFR, ERBB2, and HHLA2 (**Figure 6B**). Thus, immunotherapy and targeted therapy for gastric cancer patients could be suggested individually according to the risk model. Furthermore, apoptosis-regulated molecules were analyzed. Consistently, the high-risk group had lower activity of cell apoptosis (**Figure 6D**). Results of immune analysis based on the TCGA validating set were consistent with GEO (**Supplementary Figure S5a–e**). To be concluded, the risk model based on the Lauren type was predictive and accurate. Patients with high risk had similar features to diffuse-type patients, including more immune cells but fewer functions, inhibited cell apoptosis, better antigen recognition, and potential better immunotherapy efficiency. This Lauren-based risk model possibly provided helpful information to achieve individual treatment for GC patients.

### Construction of nomogram model

**Figure 7:**
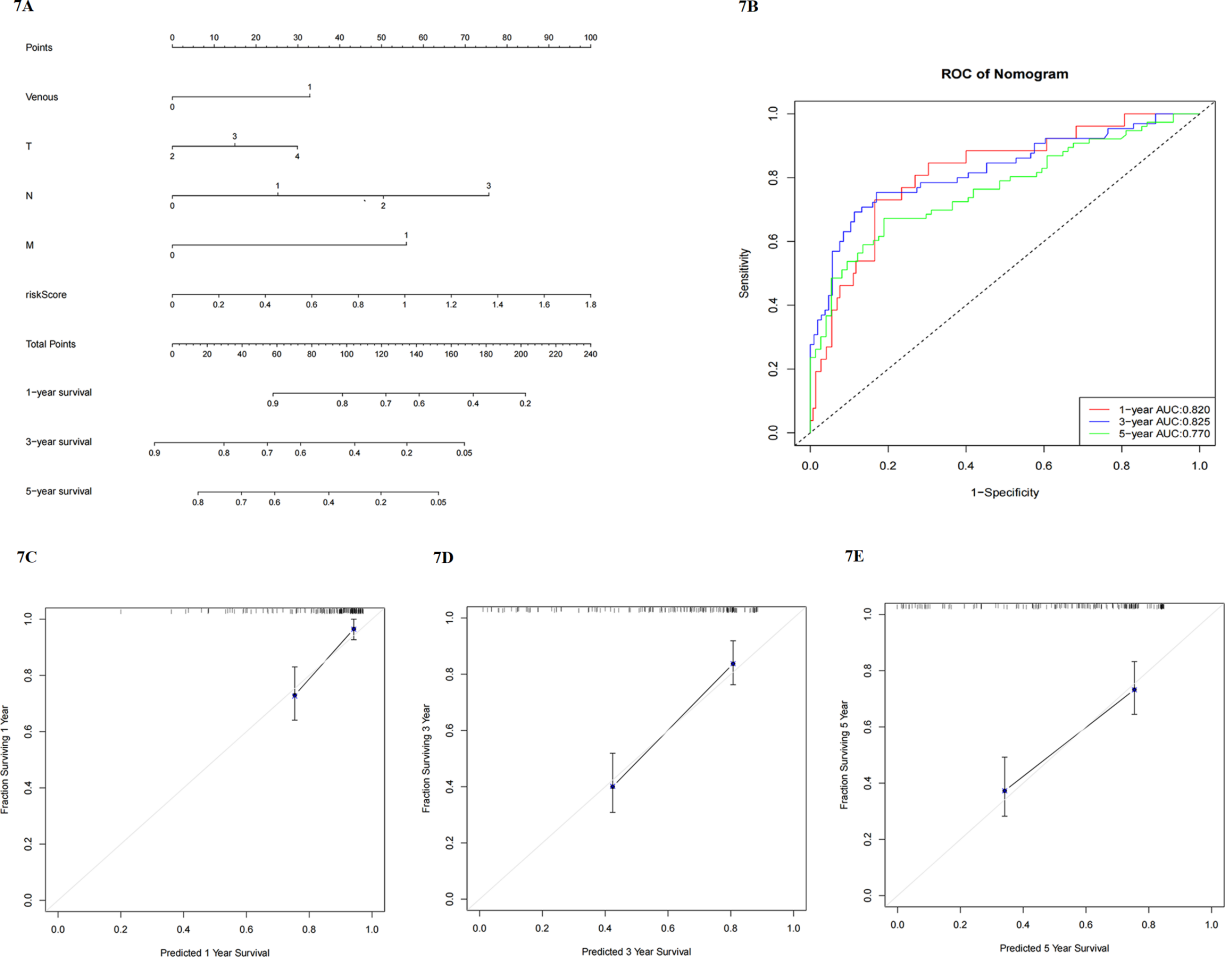
**(A)** nomogram model including clinicopathological factors and risk score; **(B)** ROC to analyze the predictive efficiency of nomogram model; **(C–E)** calibration plots of 1-year, 3-year, and 5-year survival for nomogram model.

A nomogram model was constructed based on multivariate COX regression, including risk score and clinical traits, such as venous invasion, T, N, M stage, and Lauren-related regulator signature. Using the nomogram model, clinicians could predict the 1-year, 3-year, and 5-year survival probability (**Figure 7A**). Each patient would receive a total point through the calculation, and those with higher scores had a poorer prognosis. The calibration plots indicated that the performance of this nomogram model was not inferior to the ideal model (**Figures 7C–E**). Furthermore, ROC curves were used to assess the predictive ability of the nomogram. 1-year, 3-year, and 5-year AUC values were 0.820, 0.825, and 0.770 respectively. Therefore, this model was better predictive for the Prognosis of GC patients (**Figure 7B**).

## Discussion

As per many reports, Lauren type is a potential predictive factor for GC on account of the following reasons (3, 5, 6). Firstly, the Lauren type is conserved and would not change with the development of the tumor (4). Then, it can be detected at the early stage of GC via pathological examinations. Next, it has been revealed in meta-analysis and studies that Lauren type is significantly correlated with the prognosis of GC patients (3, 18). Furthermore, Lauren type correlates with aggressive features of tumor cells and disease progression in GC (19, 21). Diffuse-type tumor, which is more prevalent in younger female patients, shows more malignant features, which induces more repaid disease progression, thus resulting in a worse prognosis, as proved in this study. It has been confirmed that diffuse-type GC patients have a higher risk of peritoneal metastasis, leading to malignant ascites, which is also consistent with the findings of this study (2). According to this study, diffuse-type GC patients are more prone to suffer from peritoneal and lymphatic vessel invasion, and they have more positive lymph nodes and advanced pathological stages. It can be found that there are differences in the sensitivity to chemotherapy and radiotherapy between different Lauren subtypes (7–11, 22, 23). Hence, it is required to select personalized treatment for patients according to their Lauren types, so as to prolong their survival. It can be speculated that aggressive features of diffuse-type tumors and discrepancies in efficacy are closely correlated with the TIM. However, there is still a lack of detailed and sufficient information for deep analysis. This study aims to compare the differences in immunity between Lauren subtypes, provide possible treatment suggestions based on Lauren subtypes, and find the key genes that potentially caused these differences.

IHC staining for immune checkpoints was performed on 360 GC specimens, and the results showed that the expressions of CD3, CD8 and CD57 were higher in diffuse-type GC than in intestinal-type one. CD3, as a molecular marker of T cell, presents in T cell and binds to T cell receptor (TCR) to determine the immune response. Highly expressed CD3 in the diffuse group presented more infiltrated T cells in their immune microenvironment, providing the potential therapeutic targets. Improvement of CD3-TCR complex promoted tumor immune activity (24–26). Thus, bispecific antibodies based on CD3-TCR have become a hot spot in the research of tumor targeting therapy recently, especially anti-CD3/CD20 therapy for lymphoma and anti-CD3/EpCAM for GC (26–28). CD3-based bispecific antibodies have the potential to improve the survival in patients with diffuse-type GC. T cells expressing CD8 coreceptor were considered to play a crucial role in adoptive immunity. With the assistance of DCs and CD4^+^ T cells, CD8+ T cells exerted a defensive function in TME to suppress tumor progression (29–31). However, recent studies reveled that CD8 T cells in diffuse-type GC were nonfunctional (19), and more details would be further analyzed in subsequent studies. CD57, as a T cell senescence marker, is expressed in nonfunctional T cells (32, 33). Immunosenescence, caused by thymus degeneration (34–37), mostly occurs among the elderly, not only affecting the innate immune system, but also reducing the function of acquired immune system. Recent studies found that in the process of immunosenescence, CD8+ T cells with lower toxicity played a pivotal role in tumorigenesis and cancer treatment (38). In this study, the patients with diffuse-type GC exhibited higher expression of CD8+ T cell and CD57, potentially indicative of T cell senescence and immunosenescence. Thus, it can be concluded that diffuse-type GC is likely to be senescent and nonfunctional despite more T cell infiltration. Compared with intestinal-type GC, diffuse-type GC might lead to tumor growth and metastasis due to immunosenescence, resulting in a lower survival. To test this further, we analyzed additional RNA-seq data and clinical information from public databases.

Subsequently, a more comprehensive analysis was conducted utilizing GEO and TCGA datasets to meticulously examine the disparities in immune cell types and functions among different Lauren subtypes. It showed that diffuse-type tumors were demonstrated to present more infiltrated immune cells, but they were less functional than intestinal-type ones. HLA-II family, especially HLA-DP, HLA-DQ, and HLA-DR, expressed on the surface of antigen-presenting cells (APC) and activated T cells, which were responsible for antigen presentation and activation for CD4 T cells (39, 40), were upregulated in diffuse-type tumors. The upregulated APCs were possibly correlated with the increased expression of HLA-II. However, the immune analysis showed that most upregulated APCs in diffuse-type tumors were non-functional. As a result, a similar level of CD4 T cells was found in groups. Notably, CD8 T cells were more activated in diffuse-type tumors. To the best of our knowledge, CD8 T cells were the primary immune cells for killing cancer cells that presented HLA-I molecules (18, 19). CD8 T cells would first be primed with the assistance of CD4 T cells and DCs, and then activated to become the effector cytotoxic T lymphocytes (CTLs) (40, 41). With the progression of tumors, the killing function of CTLs was limited by immunosuppressive barriers, which were composed of tumor stromal cells, such as cancer-associated fibroblasts (CAFs), type 2 (M2) macrophages, and regulatory T cells (Tregs). Additionally, CTL-associated antigen 4 (CTLA-4) and programmed death-1 receptor-ligand (PD-L1) would be upregulated to inhibit immune responses (42, 43). In this study, we found that the enrichment of CD4 T cells, activated DCs, and HLA-I molecules in diffuse-type tumors was similar with that in intestinal-type ones. Thus, CD8 T cells were not overactivated in diffuse-type tumors compared with those in intestinal-type ones. Moreover, the diffuse subgroup presented a higher level of stromal scores, indicating that they had more stromal cells than intestinal-type ones. CAFs, responsible for mediating the immunosuppressive TME and most abundant in the tumor stromal (44, 45), were potentially upregulated in diffuse-type tumors. In addition, tumor-promoting M2 polarization was more common, and immune-suppressed checkpoints like CTLA-4 presented higher expression in diffuse-type tumors. Thus, diffuse-type tumors were more likely to be immunosuppressed. Further, apoptosis was inhibited in diffuse-type tumors due to the reduced expression of apoptosis-related genes. In summary, although CD8 T cells showed higher expression in diffuse-type tumors, they were still less functional, which was consistent with IHC staining. The tumor constructed a more substantial immunosuppressive barrier correspondingly, leading to a lower level of immunity, more aggressive features of diffuse-type tumor cells, and a worse prognosis of patients. Results of Li et al. were consistent with this study (19). Results of IHC staining and immune analysis demonstrated that lower immunity in diffuse-type GC was possibly attributed to immunosenescence and substantial immunosuppressive barrier. Nevertheless, the immune modulatory mechanisms of Lauren subtypes remain unclear. Therefore, it is necessary to conduct further explorations.

The low efficiency of immunotherapy is mainly attributed to multidrug resistance. T cell-based immunity in the TME plays a crucial role in transferring immunoresistance to the immunostimulatory profile for GC (46–48). As revealed in some studies, CTL-high patients showed better responses to immunotherapy than CTL-low ones (47). The apoptosis analysis results showed that most apoptosis-regulated genes were inhibited except for Fas and granzymes A (GZMA), which were the direct and central pathways of CTL for killing target cells. Thus, as proved by checkpoints-based analysis, diffuse-type tumors with high expression of CTLs presented sensitivity to immunotherapy. Checkpoints, such as CD27, CD40, CD86, and CTLA4, were upregulated in diffuse-type tumors, while ERBB2 (HER2) was highly expressed in intestinal-type tumors. According to the different expression levels of checkpoints, immunotherapeutic strategies can be formulated individually. Notably, combined immunotherapeutic strategies instead of one approach alone are more effective in stimulating CTL-related immunity (41). Furthermore, immunotherapy is always considered a supplementary treatment to chemotherapy and radiotherapy in clinical practice. According to several reports, diffuse-type GC patients presented higher efficacy and less resistance to drugs, including S-1, irinotecan, and docetaxel, compared with intestinal-type patients (7–11). On that basis, personalized treatment shall be made based on pathological classification. Additionally, ARTIST I revealed that adjuvant chemoradiotherapy may be beneficial to intestinal-type GC patients (HR for OS: 0.442; 95% CI 0.493-0.994), but nonsignificant for diffuse-type ones (22), which was consistent with the findings of Ma and Mansouri (49, 50). According to some studies and meta-analyses, additional radiotherapy can improve the prognosis of patients mainly through decreasing locoregional recurrence (22, 49, 51–53), However, the worse prognosis of diffuse-type GC patients resulted from their higher risk of distant metastasis. It can be concluded that chemotherapy combined with immunotherapy should be recommended for diffuse-type GC patients, while chemoradiotherapy combined with HER2-targeted therapy is more conducive to intestinal-type patients. Consistently, some previous studies indicated that the HER2-positive rate was higher among intestinal-type GC patients compared with diffuse-type ones (54–56). Identifying the molecular subtypes of GC plays a vital role in the application of personalized medicine.

To find the hub genes possibly responsible for the differences in the tumor microenvironment between Lauren subtypes and verify the predictive efficiency of Lauren subtypes for all GC patients, we constructed a Lauren-based risk model with prognosis-related differentially expressed genes (DEGs). Three genes were included in this risk model, involving LINC00702, C8orf88, and FILP1, all upregulated in the high-risk group. GC patients could be divided into the high- and low-risk groups according to their risk scores. This risk model was independently correlated with survival, and patients in the high-risk group showed a worse prognosis than those in the low-risk group, regardless of OS and RFS. Of note, this model presented promising predictive efficiency, especially for the long-term survival of patients. Therefore, the Lauren-based risk model was proved to be effective in predicting the prognosis of GC patients. The GSEA analysis results indicated that high-risk tumors were more likely to activate tumor-promoting pathways, such as MAPK and calcium signaling pathways, and promote focal adhesion, thus contributing to tumor development and metastasis, compared with low-risk tumors. Besides, it was found through clinicopathological analysis that female patients were more prone to suffer from high-risk tumors and patients in the high-risk group showed more advanced pathological stages. It can be speculated that the activation of tumor-promoting pathways and more advanced tumor stages were possibly the leading causes of the worse prognosis of high-risk patients. Therefore, Lauren subtypes deserve more attention in clinical practice.

Three genes mainly contributing to the aggressive features of diffuse-type GC patients were included in the risk model, namely LINC00702, C8orf88, and FILP1. Long noncoding RNAs (lncRNAs) were crucial for the development and metastasis of tumors. It was reported that LINC00702 was upregulated in many types of cancers, such as ovarian cancer and meningioma, and it would accelerate the progression of these cancers (57–59). However, the functions of LINC00702 in GC were less investigated. In this study, it was found that the upregulated LINC00702 was closely related to the polarization of M2, suppression of antigen presentation, and inactivated immune cells. Consequently, LINC00702 was prominent in malignant features and immunosuppression of diffuse-type tumors, and hence further explorations into LINC00702 were also required. FILIP1 was responsible for cell migration and motility, as proved by numerous studies (60, 61). Filamin A, whose expression was closely related to FILIP1, was correlated with T cell activation and membrane rearrangement (62). Thus, FILIP1 was possibly correlated with the regulation of T cell-based immunity. Chromosome 8 open reading frame 88 (C8orf88) was included in a model to predict the survival of prostate cancer patients (63). In this study, it was demonstrated that the expression of C8orf88 was negatively correlated with immune cells and anti-tumor cells, while it was positively correlated with immune suppression and tumor development. However, the detailed mechanisms of C8orf88 remain unclear due to insufficient information. LINC00702, C8orf88, and FILP1, upregulated in diffuse-type GC, promoted tumor progression and metastasis through immune suppression and activation of tumor-promoting pathways.

The immune analysis results based on the risk model were consistent with the Lauren-related immune profile. Compared with low-risk patients, high-risk patients with a lower level of tumor immunity were potentially more sensitive to immunotherapy, and apoptosis was less likely to occur in their tumor cells, thus leading to a worse prognosis. Conversely, low-risk patients had better tumor immunity and were more suitable to receive EGFR- or HER2-based targeted therapy. The consistent results demonstrated that the Lauren-based risk model was practical in predicting GC and effective for all patients. Subsequently, a Nomogram model was constructed to improve the predictive efficiency, including Lauren-based risk scores and crucial clinicopathological features. This model was validated to be effective in predicting the long-term survival of GC patients. It can be obtained that the different expression levels of LINC00702, C8orf88, and FILP1 significantly contributed to the differences in immunity between Lauren subtypes, providing the research direction in the future. Further experimental validations are required in the future.

There are some limitations in this study. Firstly, although we provided crucial and rational information for the immune differences between Lauren subtypes, the detailed mechanism was still far from clear. Furthermore, while multivariable Cox regression results were consistent between GEO and validation sets, the absence of key clinical covariates may limit model comparability. Ongoing clinical studies at our institution will provide further validation. Lastly, it could be proved that Lauren subtypes were correlated with the sensitivity to immunotherapy and targeted therapy, whereas there was a lack of clinical validation. It is required to conduct further experimental validations *in vivo* or *in vitro*. Thus, more investigations are needed in the future.

## Conclusion

Lauren subtypes can be used to predict the prognosis of GC patients and they were closely correlated with tumor immunity. Diffuse-type tumors had more abundant immune cells but fewer functions which may be contributed to immunosenescence, and they were potentially more sensitive to immunotherapy and chemotherapy than intestinal-type tumors. It can be strongly recommended that intestinal-type GC patients could receive HER2-targeted therapy and chemoradiotherapy. These differences can be mainly attributed to upregulated LINC00702, C8orf88, and FILP1 in diffuse-type GC patients. This Lauren-based risk model was validated to be effective in predicting the survival of GC patients.

## Data Availability

The datasets presented in this study can be found in online repositories. The names of the repositories and accession number(s) can be found within the article.
