# Supplementary figures and images for: Immune analysis according to Lauren type for gastric cancer and its significance in individual treatment and prognostic prediction
Source: Front Immunol. 2025 Jul 24;16:1589513. doi: 10.3389/fimmu.2025.1589513 (PMC12328303; doi:10.3389/fimmu.2025.1589513)

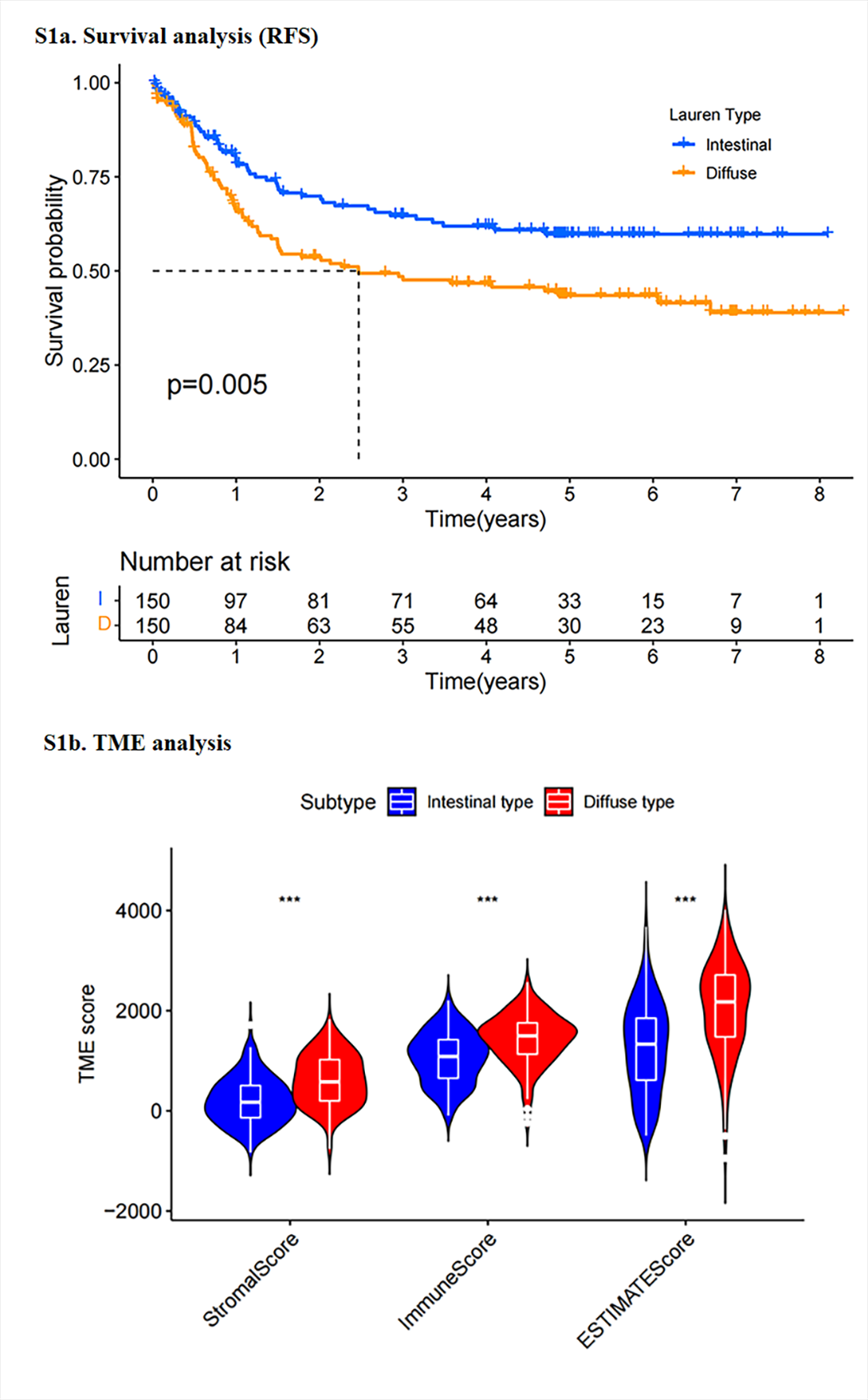

Supplement: Supplementary file 1 [file Image1.tif]

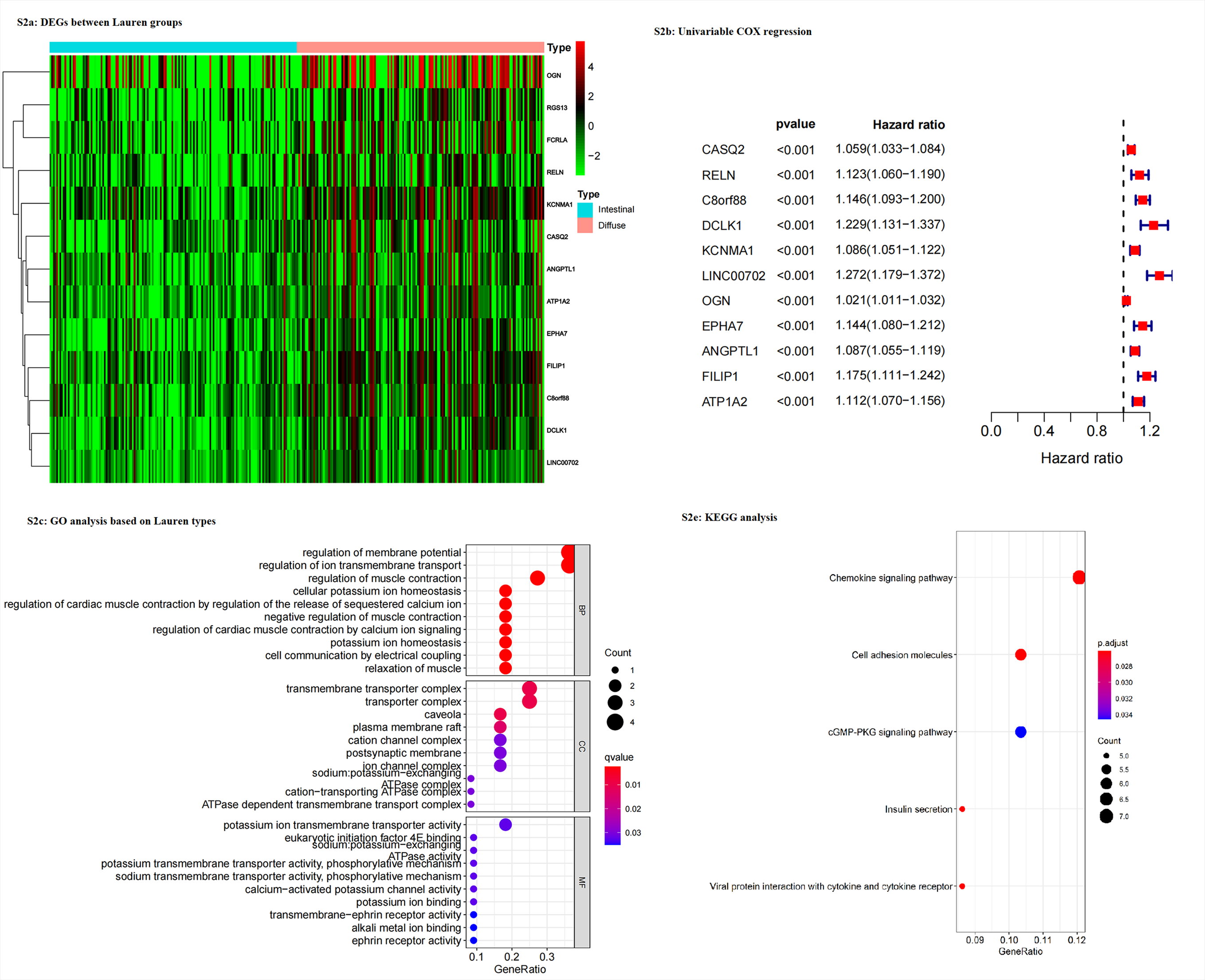

Supplement: Supplementary file 2 [file Image2.tif]

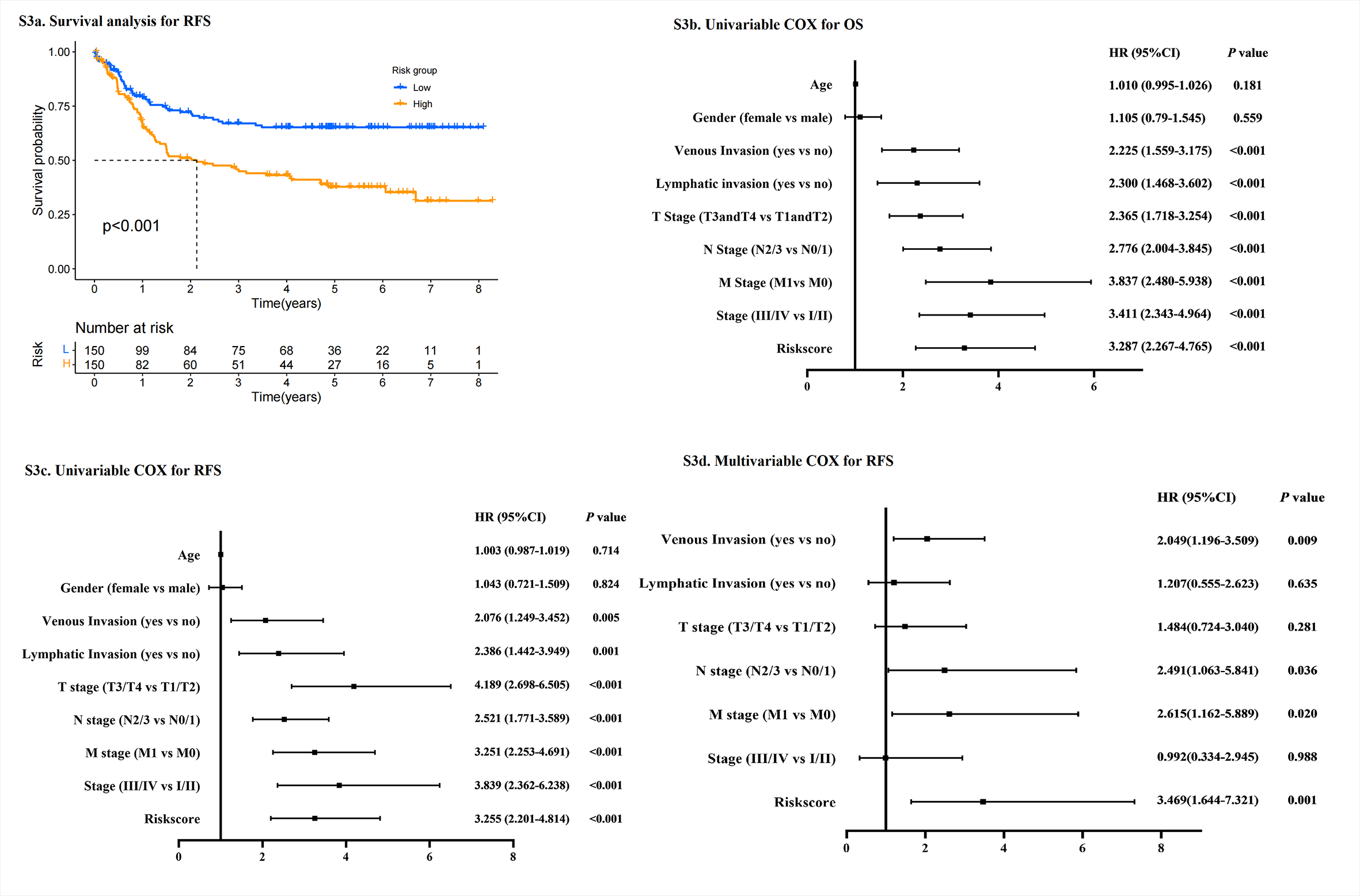

Supplement: Supplementary file 3 [file Image3.tif]

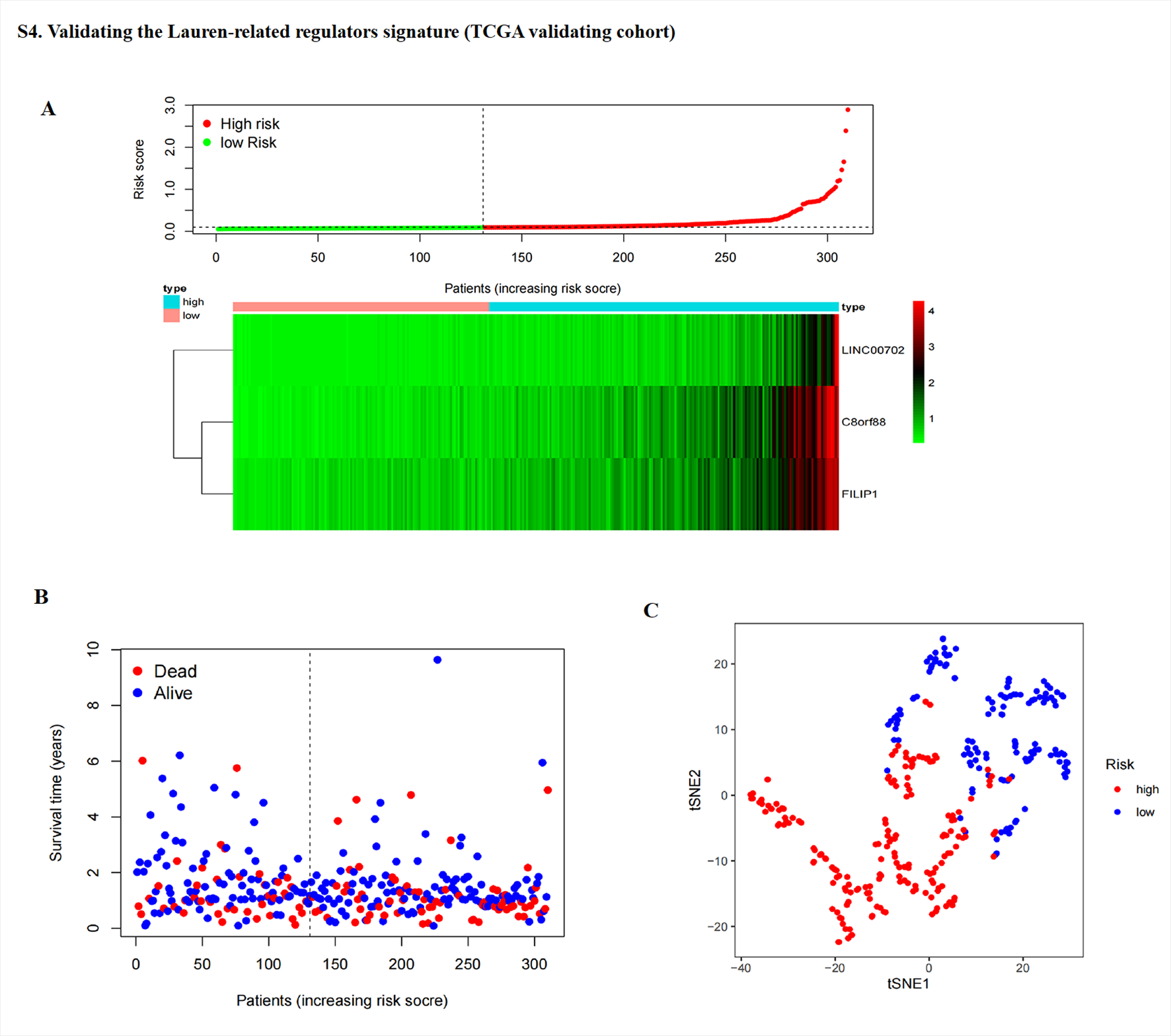

Supplement: Supplementary file 4 [file Image4.tif]

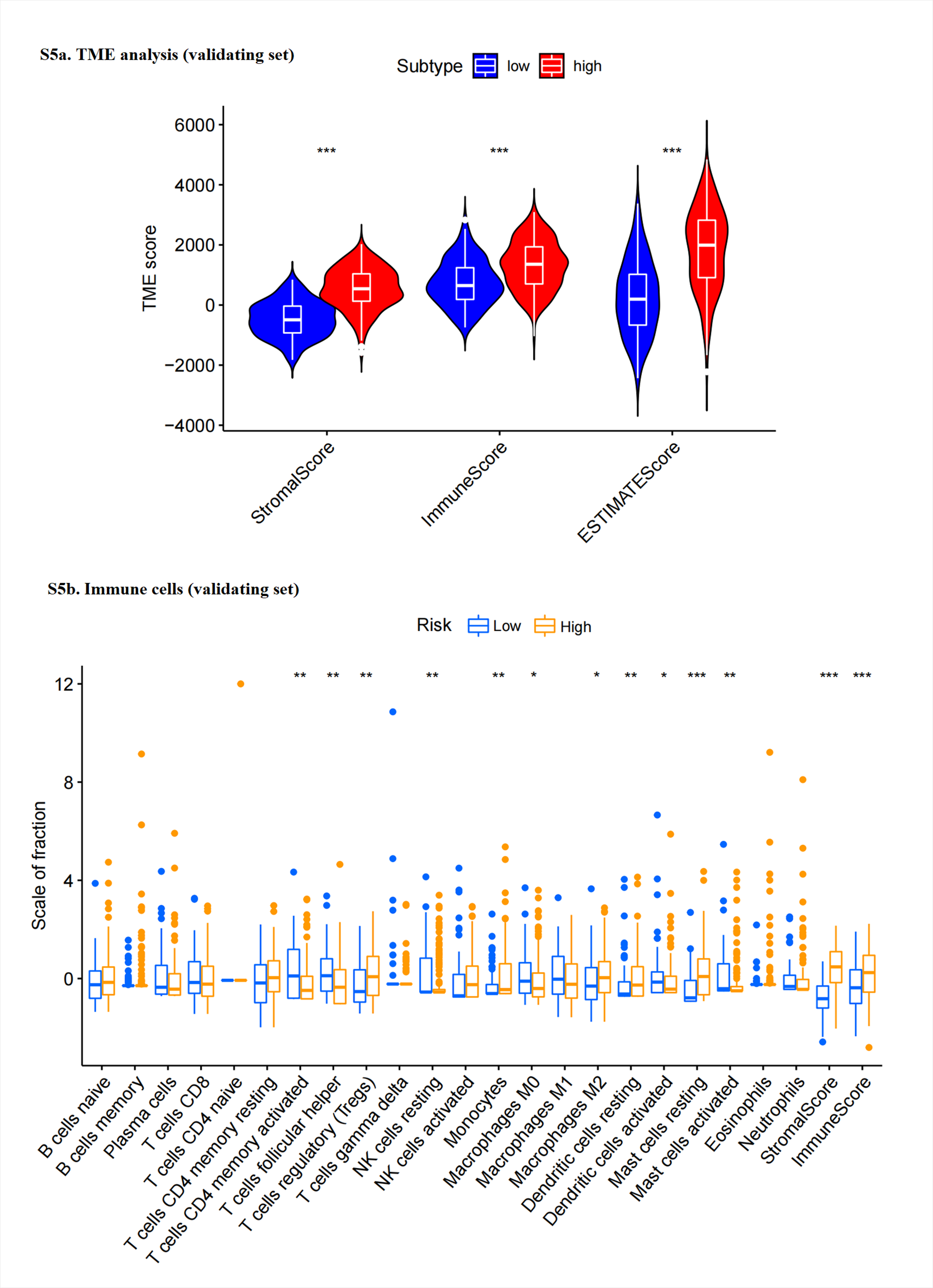

Supplement: Supplementary file 5 [file Image5.tif]

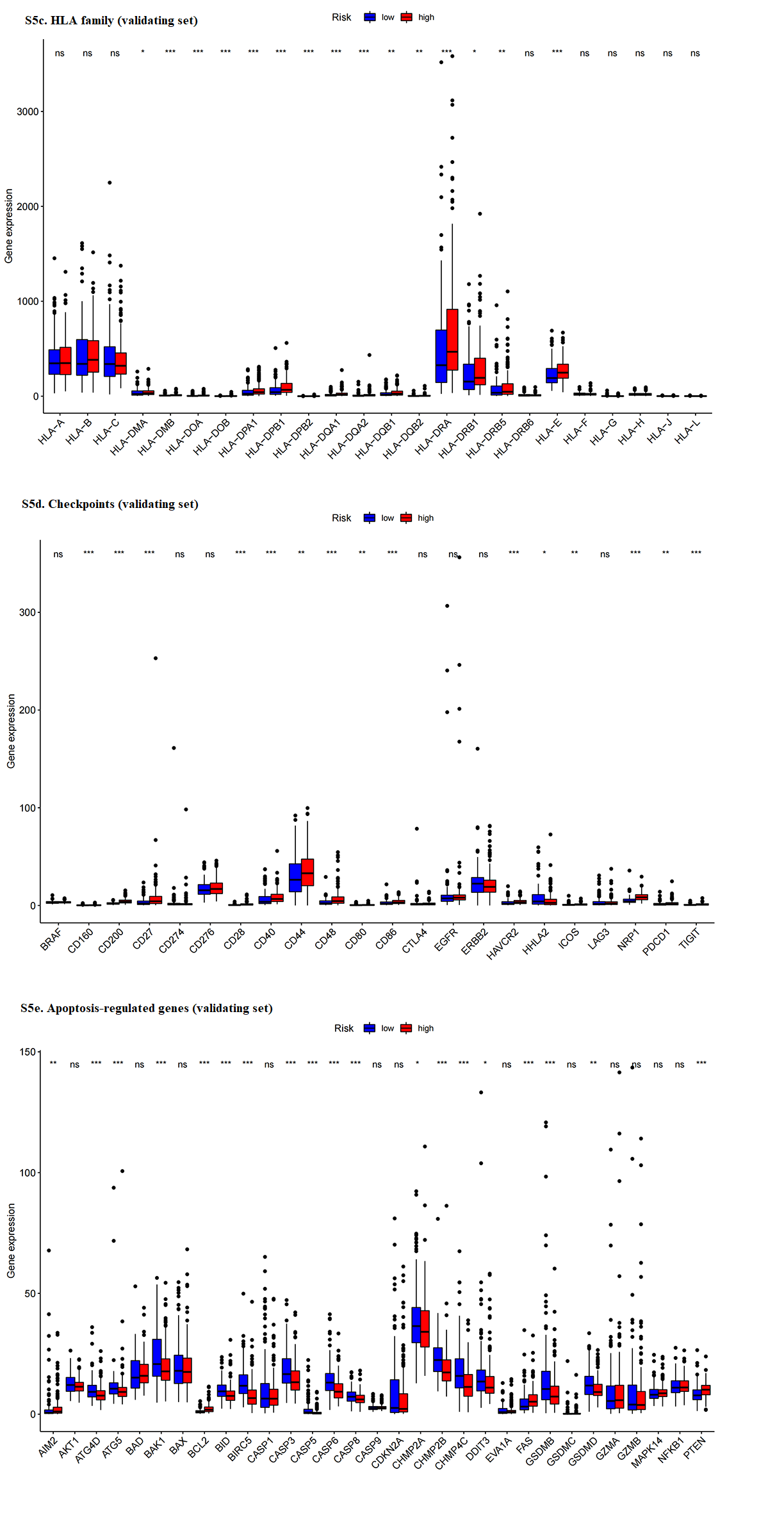

Supplement: Supplementary file 6 [file Image6.tif]
