# Supplementary material for: Immune analysis according to Lauren type for gastric cancer and its significance in individual treatment and prognostic prediction
Source: Front Immunol. 2025 Jul 24;16:1589513. doi: 10.3389/fimmu.2025.1589513 (PMC12328303; doi:10.3389/fimmu.2025.1589513)
Supplement: Supplementary file 7 [file Table1.docx]

**Table S1: Differentially expressed genes between Lauren groups**

| gene | conMean | treatMean | logFC | pValue | fdr |
| --- | --- | --- | --- | --- | --- |
| CASQ2 | 0.894049 | 3.711857 | 2.053716 | 8.59E-06 | 7.17E-05 |
| RGS13 | 0.770484 | 2.287198 | 1.569745 | 3.31E-07 | 5.07E-06 |
| RELN | 0.74652 | 2.13647 | 1.516977 | 3.31E-06 | 3.30E-05 |
| C8orf88 | 0.884971 | 2.554293 | 1.529223 | 4.21E-10 | 2.74E-08 |
| DCLK1 | 0.521491 | 1.529116 | 1.551983 | 1.35E-08 | 3.94E-07 |
| KCNMA1 | 1.240441 | 3.622407 | 1.546096 | 1.08E-10 | 9.77E-09 |
| LINC00702 | 0.424752 | 1.266936 | 1.576652 | 9.49E-09 | 2.92E-07 |
| FCRLA | 0.968604 | 3.138856 | 1.696261 | 6.76E-06 | 5.87E-05 |
| OGN | 4.667458 | 13.31329 | 1.512159 | 5.47E-07 | 7.58E-06 |
| EPHA7 | 0.650821 | 1.984235 | 1.60825 | 3.75E-08 | 8.82E-07 |
| ANGPTL1 | 0.990831 | 3.128589 | 1.658801 | 4.57E-05 | 0.000283 |
| FILIP1 | 0.881213 | 2.502437 | 1.50577 | 2.08E-12 | 5.58E-10 |
| ATP1A2 | 0.657293 | 2.261697 | 1.782798 | 2.73E-06 | 2.81E-05 |
