# Supplementary material for: Immune analysis according to Lauren type for gastric cancer and its significance in individual treatment and prognostic prediction
Source: Front Immunol. 2025 Jul 24;16:1589513. doi: 10.3389/fimmu.2025.1589513 (PMC12328303; doi:10.3389/fimmu.2025.1589513)
Supplement: Supplementary file 8 [file Table2.docx]

Risk score =$\sum_{i=1}^{n} \left( coefi*expri \right)$

The variable coef_i_ represented the lasso regression coefficients of gene I, and expr_i_ was referred to the relative expression of genes for patients i. The median risk score of the GEO training set was considered the cut-off point. Then patients in training and validating sets were then divided into low- and high-risk groups using the same cut-off point. The associations between risk groups and survival were analyzed via the Kaplan-Meier curve and log-rank test. Time-dependent receiver operating characteristic (ROC) curve and the principal component analysis (PCA) were analyzed by ROC and Rtsne packages respectively, to validate the accuracy of survival prediction of this risk model.
